# Evaluation of Ki67 Expression across Distinct Categories of Breast Cancer Specimens: A Population-Based Study of Matched Surgical Specimens, Core Needle Biopsies and Tissue Microarrays

**DOI:** 10.1371/journal.pone.0112121

**Published:** 2014-11-06

**Authors:** Gøril Knutsvik, Ingunn M. Stefansson, Sura Aziz, Jarle Arnes, Johan Eide, Karin Collett, Lars A. Akslen

**Affiliations:** 1 Centre for Cancer Biomarkers CCBIO, Department of Clinical Medicine, Section for Pathology, University of Bergen, Bergen, Norway; 2 Department of Pathology, Haukeland University Hospital, Bergen, Norway; University of Torino, Italy

## Abstract

**Introduction:**

Tumor cell proliferation in breast cancer is strongly prognostic and may also predict response to chemotherapy. However, there is no consensus on counting areas or cut-off values for patient stratification. Our aim was to assess the matched level of proliferation by Ki67 when using different tissue categories (whole sections, WS; core needle biopsies, CNB; tissue microarrays, TMA), and the corresponding prognostic value.

**Methods:**

We examined a retrospective, population-based series of breast cancer (*n* = 534) from the Norwegian Breast Cancer Screening Program. The percentage of Ki67 positive nuclei was evaluated by visual counting on WS (*n* = 534), CNB (*n* = 154) and TMA (*n* = 459).

**Results:**

The median percentage of Ki67 expression was 18% on WS (hot-spot areas), 13% on CNB, and 7% on TMA, and this difference was statistically significant in paired cases. Increased Ki67 expression by all evaluation methods was associated with aggressive tumor features (large tumor diameter, high histologic grade, ER negativity) and reduced patient survival.

**Conclusion:**

There is a significant difference in tumor cell proliferation by Ki67 across different sample categories. Ki67 is prognostic over a wide range of cut-off points and for different sample types, although Ki67 results derived from TMA sections are lower compared with those obtained using specimens from a clinical setting. Our findings indicate that specimen specific cut-off values should be applied for practical use.

## Introduction

Breast cancer is a heterogeneous disease. During the last decade, gene expression studies have identified distinct molecular subtypes, such as Luminal A, Luminal B, HER2-enriched, basal-like and normal breast-like, and these have markedly different behavior and prognosis [1], [2]. Subsequent studies have introduced immunohistochemical surrogate markers for molecular classification, with a proposed Ki67 cut-point of 14% to separate Luminal A from Luminal B tumors [3], [4]. Furthermore, the treatment effect of adding docetaxel to highly proliferative, luminal tumors has been demonstrated [5], [6].

In 2011, the St Gallen International Expert Consensus included a Ki67 cut-off point of 14% in their recommendations for adjuvant therapy [7]. However, there is currently no agreement on specimen selection, technical protocols, evaluation methods or cut-off values [8], [9], and the criteria for sub-classification of breast carcinomas by Ki67 has yet to be established. This area is controversial, and in the report from St Gallen 2013 recently published, the cut-off value has been changed [10].

On this background, we aimed to study the levels of tumor cell proliferation based on Ki67 expression according to specimen type such as whole sections (WS), core needle biopsies (CNB) and tissue microarrays (TMA) from a population-based series of breast cancers, and to study and compare the prognostic value of Ki67 in relation both to specimen type and molecular subgroups of breast cancer.

## Materials and Methods

### Patient series

This study was approved by the Western Regional Committee for Medical and Health Research Ethics, REC West (REK 2012/1704). We identified all women (50–69 years) who resided in Hordaland County, Norway, when diagnosed with primary invasive breast cancer as part of the population-based Norwegian Breast Cancer Screening Program during 1996-2003. Hordaland County has approximately 500,000 inhabitants, this represents about 10% of the total population of Norway.

Patients with distant metastatic disease at time of diagnosis (stage IV) were not included, leaving 555 potential cases. Written informed consent was not obtained from the patients, but in accordance with national ethics guidelines and procedures for such retrospective studies, all participants were contacted with written information on the study and asked to respond if they objected. In total, 9 patients (1.6%) did not approve participation. 12 cases had technical inadequate material for proliferation assessment (Ki67), leaving 534 cases for further studies. Patient records and information were anonymized and de-identified prior to analysis. The patients included had a median age of 60 years at diagnosis (factual range 49–72 years).

The patients received treatment according to standard national protocols in a single institution. Follow-up information was given by the Norwegian Cause of Death Registry, and can be considered accurate and complete. Last date of follow-up was December 31, 2011. Outcome data include survival status, survival time and cause of death. During the follow-up period, 79 patients (15%) died from breast carcinoma, and 62 (12%) died from other causes. The median survival of the censored patients was 12 years, and the median follow-up was 13 years calculated by the reverse Kaplan-Meier method. The 5-year breast cancer specific mortality was 9% (49/534).

### Clinico-pathological variables

Patient's clinical history and tumor characteristics including age at diagnosis, largest tumor diameter, histologic type, histologic grade, lymph node status and hormonal receptor status were obtained from the clinical records and routine histopathology reports. Histologic type was assessed according to WHO criteria, whereas histologic grade was evaluated using the Nottingham modification [11] by five experienced breast pathologists (JE, JA, IMS, KC, LAA). Tumor size was assessed histologically (61%) and by macroscopic examination (29%). However, if pathologic tumor size was not available (as in patients with locally advanced or multifocal disease), the radiologic size estimate was included (10%). For immunohistochemical studies on whole sections, HE slides were re-examined, and representative slides (1–2 blocks) displaying both the peripheral and central parts of the tumor, as well as the most cellular and high-grade areas, were selected for further analyses. The corresponding FFPE block was also used for TMA construction.

### Patient characteristics

Radical mastectomy was performed in 285 cases (53%), and breast conserving surgery in 245 cases (46%); four patients were represented with core needle biopsy only (three cases of locally advanced disease and one patient with surgery abroad). Adjuvant therapy was decided according to tumor size, histologic grade, hormone receptor status and nodal status. Treatment protocols showed slight modifications during the period. Chemotherapy was offered to patients below 55 years with stage I disease who had histologic grade 2 and 3 tumors, and to patients under 55 years with stage II disease. From 1998, chemotherapy was also recommended for patients between 55–65 years with stage I or II disease combined with hormone receptor negativity. 33 patients (6%) were treated with neo-adjuvant chemotherapy due to locally advanced disease.

Adjuvant radiation therapy was recommended for patients who received breast conserving surgery, had primary surgery without free resection margins, stage II disease with axillary metastasis, as well as stage III disease.

### Specimen characteristics

The tumor samples were fixed in 4% buffered formaldehyde before processing and embedding in paraffin. Storage time of the archival formalin-fixed, paraffin embedded tissue samples (blocks) was up to 17 years. Five µm sections were cut by one person using the same microtome and mounted onto poly-lysine coated glass slides. Slides were stored for no longer than two weeks at 4°C until staining for Ki67 was performed.

#### Tissue microarray (TMA)

H&E stained slides were used for tumor verification. Briefly, 1.0 mm cores in triplicate were punched and mounted into a recipient paraffin block using a semi-automated precision instrument (Minicore 3, Tissue Arrayer, Alphelys, France). Care was taken to select areas with high tumor purity and to include the periphery and areas of highest histologic grade. 190 cases had previously been processed [12], [13]; from these cases three tissue cores with a diameter of 0.6 mm were obtained by a different instrument (Beecher Instruments, Silver Spring, MD, USA).

Among the 534 cases with TMA available, 22 cases had tissue cores devoid of invasive tumor, 21 cases had complete core loss and 32 cases showed fewer than 100 tumor cells on arrayed spots, leaving 459 cases (86%) available for proliferation assessment.

#### Preoperative core needle biopsies (CNB)

182 patients had undergone both preoperative core needle biopsy and subsequent primary surgical excision for breast carcinoma. Among these, 25 cases were excluded due to non-representative or inadequate material remaining for biomarker assessment. Three cases had previously been excluded due to lack of informed consent. In total, 310 cases received preoperative cytology only, and the remaining cases had either frozen sections, incisional or excisional biopsies performed; this practice was according to national guidelines at the time. The number of core biopsies taken ranged from 1 to 4 (mean = 2.4, median = 2). 92% of the cases had more than 1 core biopsy available.

### Ki67 immunohistochemistry

Immunohistochemistry was performed on 5 µm slides of formalin-fixed and paraffin-embedded archival tumor tissue. The sections were de-waxed with xylene/ethanol before target retrieval in a pressure cooker (Decloaking Chamber Plus, Biocare Medical). Staining procedures were performed on a DAKO autostainer using the K4061/Envision Dual Link System (rabbit+mouse). Sections were incubated for 30 minutes at room temperature with a monoclonal rabbit antibody (M 7240, clone MIB-1, DAKO) at a 1∶100 dilution. Finally, diaminobenzidine (DAB) as chromogen for 10 minutes was followed by haematoxylin as counterstain for 3 minutes. Sections from tonsils were used as positive controls; negative controls were obtained by replacing the primary antibody with Tris-buffered saline. Controls were included in each staining run.

### Evaluation of staining

#### Hormone receptors

Results for estrogen and progesterone receptors were obtained from the routine pathology reports. Tumors were considered ER or PR positive if ≥10% of tumor nuclei stained positive, according to national guidelines during the period.

#### HER2

The established scoring system for DAKO Herceptest was used. HER2 SISH was performed on IHC 2+ cases (Ventana INFORM HER2 DNA probe staining). The 2+ cases were considered HER2 positive if the HER2/Chr17 ratio by SISH was equal to or greater than 2.0.

#### Ki67 scoring

All slides were examined and scored by one pathologist (GK), blinded to patient characteristics and outcome. The slides were evaluated using light microscopy (Leica DMLB) with an eye-piece graticule for counting at x630 magnification, roughly following the approach used by Weidner et al. [14]. Care was taken to avoid areas of intense inflammation, fibrosis, necrosis, low cellularity or poor fixation. The slides were scanned at low magnification (x100) to identify and encircle the hot-spot (HS); this was defined as the area containing the highest density of Ki67-labelled tumor cells by visual impression. The hot-spot was usually situated at the periphery of the carcinoma. Further, the cold-spot (CS), the area with the lowest density of Ki67 positive tumor nuclei, was identified. Overall, 23% of all cases (WS) showed clearly heterogeneous proliferation. In these cases, 500 tumor cells in consecutive HPFs were counted in both hot and cold spots. For tumors with homogenous proliferation, or small areas of invasive tumor, 500 tumor cells at the peripheral part of the tumor were assessed, and a single figure for Ki67 expression was recorded. Only stained tumor cells crossing horizontal grid lines were counted. Any nuclear staining regardless of intensity was considered positive.

We did not find any correlation between Ki67 expression and years of storage of the tissue blocks (data not shown). Further, we found no difference in median Ki67 expression when comparing patients with 1–2 core biopsies (CNB) available versus 3–4 core biopsies (data not shown).

In a subset of 50 cases, the slides were evaluated at a different magnification (x400), with excellent correlation between the methods (Spearman's correlation coefficient (ρ) 0.96, kappa-value 0.79, *P*<0.001 for both tests).

#### Observer agreement for Ki67 counts

Intra-observer variability was evaluated by randomly rechecking 50 cases (WS) after a period of 6 months, with excellent correlation between the 2 counts (Spearman's ρ 0.99, kappa-value 0.88). Moreover, a separate researcher (SA) assessed 50 cases across all sample categories showing good inter-observer agreement: WS specimens: Spearman's ρ 0.95; kappa-value 0.71; CNB specimens: Spearman's ρ 0.93; kappa-value 0.80; TMA specimens: Spearman's ρ 0.88; kappa-value 0.74 (*P*<0.001 for each analysis).

For assessment of Ki67 on CNB, 500 tumor cells were counted by choosing the most proliferative region if possible. For assessment of Ki67 on TMA, all available cores were assessed, and the core with the highest Ki67 score was recorded. TMA samples with fewer than 100 tumor cells were considered not interpretable.

Furthermore, an “average” tumor cell proliferation was estimated as a mean of Ki67-HS and Ki67-CS in cases of heterogeneity. In a subgroup of 25 cases, the overall average score was also directly counted on the slides in addition to the estimated average. This was obtained by counting 200 cells in each of three representative tumor areas (hot-spot, intermediate area and cold-spot). There was a strong and positive correlation between the average score obtained by counting and the estimated mean (Spearman's ρ 0.86, *P*<0.001, kappa-value 0.62, *P* = 0.001).

### Definition of molecular classes of breast cancer

Molecular classes were defined as Luminal A (ER positive and/or PR positive, Ki67<14%), Luminal B (LuminalB-HER2 negative: ER positive and/or PR positive, Ki67≥14%; LuminalB-HER2 positive: HR positive and HER2 positive regardless of Ki67), HER2 enriched (ER and PR negative, HER2 positive), and triple negative (ER negative, PR negative, HER2 negative) based on published criteria [7].

### Statistical methods

Analyses were performed using the SPSS statistical package, version 18.0 (SPSS Inc., Chicago, IL). Statistical significance was assessed at the two-sided 5% level. Non-parametric correlations were tested by the Spearman's rank coefficient. Bland and Altman analysis and Wilcoxon signed rank tests were used to compare related samples. Continuous variables not following the normal distribution were compared between two or more groups using the Mann-Whitney U or Kruskal-Wallis H-test. Continuous variables were categorized based on quartile limits, also considering the frequency distribution plot for each marker, as well as the number of events in subgroups. The Cohen's kappa measure was used to assess the agreement of two categorical scores.

For survival analyses, the end-point of interest was breast cancer specific survival (BCSS), defined as the time in months from the date of diagnosis to the date of death from breast cancer. Patients with missing data were excluded from analyses. Univariate survival analyses were performed using the product-limit procedure (Kaplan-Meier method), and differences between categories were estimated by the log-rank test, with date of diagnosis as the starting point. Patients who died from other causes were censored at the date of death. Multivariate survival analyses were conducted using Cox′ proportional hazards methods. Multivariate analyses adjusted for standard prognostic factors including tumor size, histologic grade, nodal status and age. Covariates were examined by log-log plot and by adding interaction terms to determine their ability to be incorporated in multivariate models. For continuous variables, the proportional hazard assumptions were also assessed by studying the graphs of Schoenfelds residuals.

## Results

### Clinico-pathologic characteristics of the patients

In the current study, median tumor size was 15 mm (range 3–110 mm). Table 1 gives an overview of clinico-pathological features of the complete series. See also table S1 in File S1 for a summary of clinico-pathologic characteristics in relation to molecular subclasses.

**Table 1:** Clinico-pathologic characteristics.

Characteristics	Complete series	
	*N*	(%)
**Tumor diameter**		
≤2 cm	405	75.8
>2 cm	129	24.2
**Histologic grade**		
1	218	40.8
2	226	42.3
3	90	16.9
**Nodal status**		
Negative	387	72.5
Positive	142	26.6
Missing	5	0.9
**Histologic type**		
Ductal	447	83.7
Lobular	55	10.3
Tubular	8	1.5
Mucinous	16	3.0
Medullary	4	0.7
Unclassified	4	0.7
**ER**		
Positive	451	84.5
Negative	83	15.5
**PR**		
Positive	377	70.6
Negative	157	29.4
**HER2**		
Negative	463	86.7
Positive	71	13.3

Among patients that underwent axillary node dissection, the median number of lymph nodes sampled was 11 (range 1–33).

### Ki67 counts in relation to different specimen types

The following results are based upon hot-spot counts, unless otherwise is stated. The median percentages of Ki67 expression according to specimen types for both the complete series and paired cases are listed in Table 2, see also Figure S1-A. Ki67 counts were significantly higher in WS as compared to CNB (n = 154, Wilcoxon signed rank test, *P* = 0.001), with a median absolute difference of 2.4% (range −44% to 42%), see Figure S1-B.

**Table 2:** Ki67 counts according to tissue categories.

Complete series	*N*	Median	Range	Mean
Ki67-WS	534	18	1–94	24
Ki67-CNB	154	13	0.4–89	17
Ki67-TMA	459	7	0.2–83	12
**Paired cases**				
Ki67-WS	137	17	0.8–90	21
Ki67-CNB	137	13	0.4–89	18
Ki67-TMA	137	6	0.2–71	10

WS, whole sections; CNB, core needle biopsies; TMA, tissue micro arrays.

Ki67 counts were significantly higher in WS as compared to TMA (n = 459, Wilcoxon signed rank test, *P*<0.001), with a median absolute difference of 10% (range −6 to 76%). Furthermore, an increase in variability of the differences with increasing proliferation was shown (See Figure S1-C).

In 137 cases with matched WS, CNB and TMA samples, the median percentages of Ki67 expression were significantly different with 17% (WS), 13% (CNB) and 6% (TMA), Wilcoxon signed rank test, *P*<0.001 for each analysis. Still, The Ki67 values obtained on WS were significantly correlated with both CNB (Spearman's ρ 0.56, *P* = 0.001) and TMA (Spearman's ρ 0.81, *P*<0.001). Further, Ki67 counts on CNB were significantly correlated with TMA (Spearman's ρ 0.49, *P*<0.001) (See Figure S2).

Using the 14% Ki67 threshold on the entire series (n = 534), based on WS specimens, 61% of tumors were classified as having high proliferation. In the CNB series (n = 154), 48% showed high Ki67 expression, as compared to 25% of the cases when using TMA specimens (n = 459), as illustrated in Figure S3.

### Associations between Ki67 and clinico-pathological features

High Ki67 expression by all 3 classes of specimens was significantly associated with high histologic grade and hormone receptor negativity (Table 3). Furthermore, elevated Ki67 expression on WS and TMA was associated with large tumor size, lymph node metastasis, and HER2 positivity. No associations were found between high Ki67 and age or tumor type. For cold-spot counts on full sections, the associations between Ki67 and tumor size and HER2 status were not significant (See Table S2 in File S1).

**Table 3:** Associations between Ki67 expression and histopathological features.

	Ki67-WS (n = 534)		Ki67-CNB (n = 154)		Ki67-TMA (n = 459)	
Variables	Median (%)	*P*-value^a^	Median (%)	*P*-value^a^	Median (%)	*P*-value^a^
**Tumor diameter**		<0.001		0.089		<0.001
≤2 cm	16.8		11.2		6.2	
>2 cm	28.0		16.8		11.2	
**Histologic grade**		<0.001		<0.001		<0.001
1	12.0		9.2		4.4	
2	19.5		14.1		7.0	
3	43.7		40.0		23.4	
**Nodal status** b		0.002		NS		0.002
Negative	16.8		11.9		6.2	
Positive	23.3		14.4		8.7	
**ER**		<0.001		<0.001		<0.001
Positive	16.6		11.0		6.0	
Negative	42.8		40.0		19.0	
**PR**		<0.001		0.005		<0.001
Positive	16.8		11.1		6.0	
Negative	26.2		19.3		12.0	
**HER2**		<0.001		0.088		<0.001
Negative	16.8		11.7		6.0	
Positive	32.4		18.4		15.2	

NS, not significant.

a Mann-Whitney or Kruskal-Wallis tests.

b 5 cases (WS), 1 case (CNB) and 4 cases (TMA) with unknown lymph node status were excluded.

### Tumor cell proliferation in different molecular subgroups

**Figure 1:**
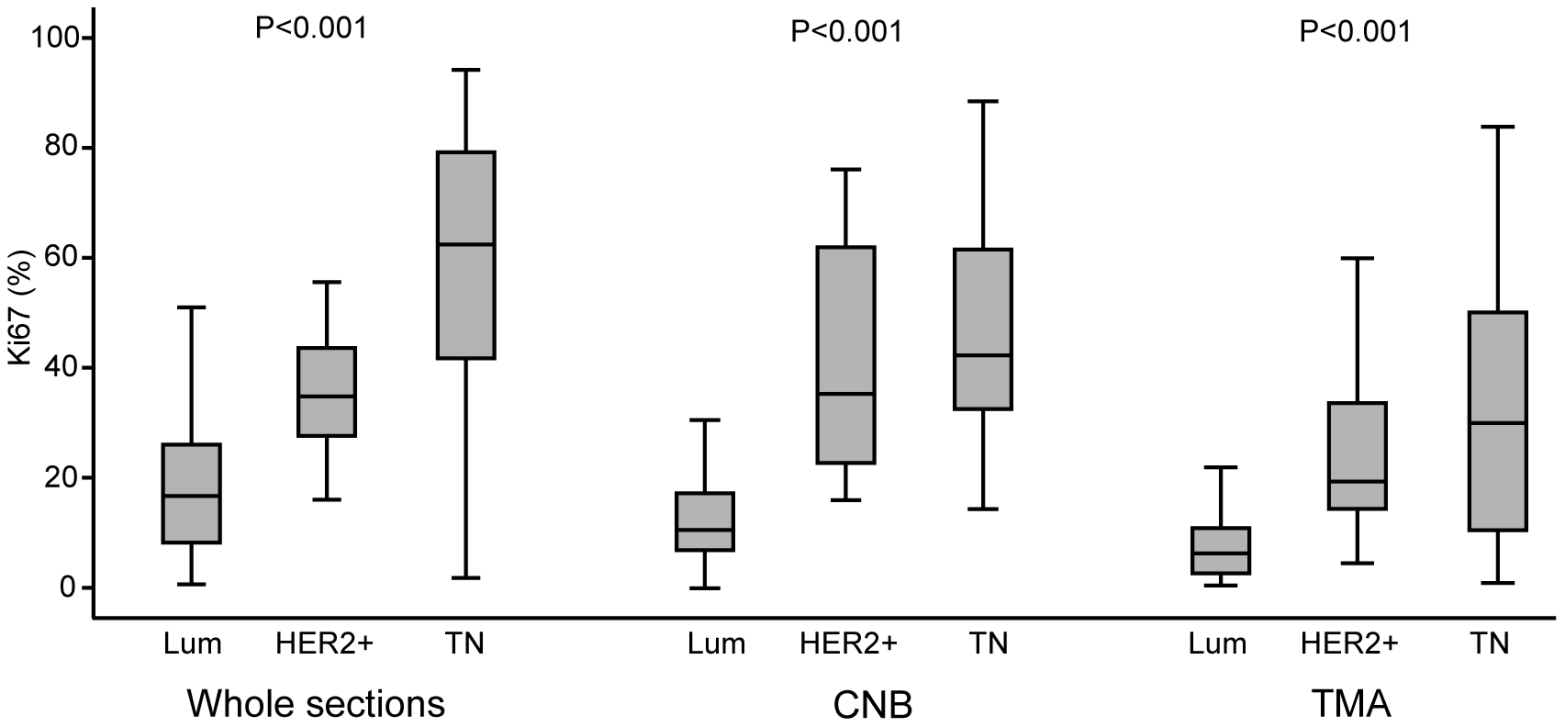
Box plots of tumor cell proliferation by Ki67 expression according to breast cancer molecular subgroups in different specimen categories. Horizontal lines inside the boxes represent the median value; box limits indicate the 25th and 75th percentiles; whiskers extend 1.5 times the interquartile range from the 25th and 75th percentiles.

Based on WS (complete series), the median expression of Ki67 in the Luminal subclass (including Luminal-HER2+) was 17% (Luminal A subclass 7%, Luminal B subclass 25%). In the HER2+ subclass (HR-, HER2+), the median expression of Ki67 was 35% whereas the triple negative subgroup demonstrated the highest Ki67 median of 62% (Kruskal-Wallis test, *P*<0.001, Figure 1). Assessment on CNB and TMA revealed the same pattern with highest proliferation shown for the triple negative group followed by the HER2+ subgroup. The lowest proliferation was observed in the Luminal subgroup.

**Figure 2:**
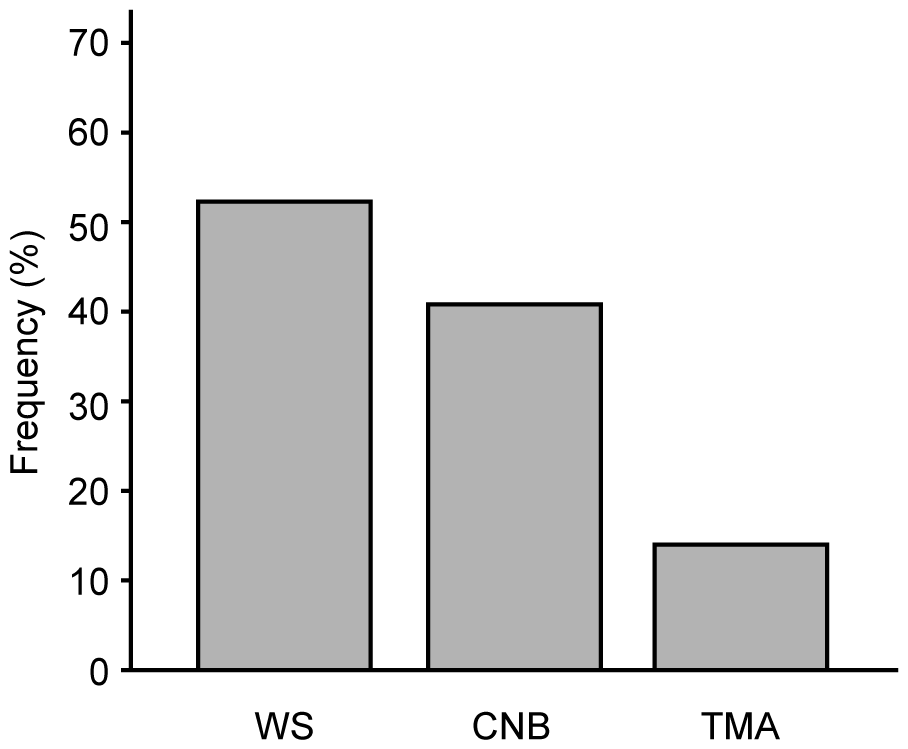
Frequency of cases in the Luminal/HER2- subgroup showing high proliferation when applying a Ki67 cutoff-point of 14% to different specimen categories. WS (*n* = 415), CNB (*n* = 125), TMA (*n* = 350).

We then applied the 14% cut-off point to WS, CNB and TMA. Among hormone receptor positive cases, excluding Luminal B/HER2+, the following figures for the frequency of cases having high proliferation were 52% (WS), 41% (CNB) and 14% (TMA), as illustrated in Figure 2. In the study by Cheang and colleagues, the Luminal B category comprised 36% of the HR+/HER2 negative cases [3]. By applying this frequency to our series, the following cut-off points for Ki67 would result in a similar size of the Luminal B (HER2 negative) subgroup: 20% (WS), 15% (CNB) and 8% (TMA).

We further applied the 14% cut-off point (St Gallen 2011) in the Luminal subgroup (excluding Luminal B/HER2+) and found classification agreement in 65% of the cases when comparing WS and CNB (n = 125, paired cases) as illustrated in Table 4 (kappa-value 0.29, *P*<0.001). Of note, 18 cases (14%) initially categorized as luminal B on CNB were downgraded on WS, whereas 26 cases (21%) categorized as luminal A on CNB were upgraded. We then compared the results between WS and TMA (n = 350, paired cases) and found concordance in 59% of cases (kappa-value 0.23, *P*<0.001). 143 cases (41%) categorized as luminal A on TMA were upgraded on WS, whereas only 1 case showed the opposite pattern.

**Table 4:** Ki67 concordance between WS, CNB and TMA in the luminal subgroup.

	WS		Agreement	Kappa	*P*-value
	LumA	LumB			
	*N* (%)	*N* (%)			
**CNB**					
LumA	48 (38)	26 (21)	65%	0.29	0.001
LumB	18 (14)	33 (26)			
**TMA**					
LumA	158 (45)	143 (41)	59%	0.23	<0.001
LumB	1 (0.3)	48 (14)			

### Tumor cell proliferation and patient outcome

**Figure 3:**
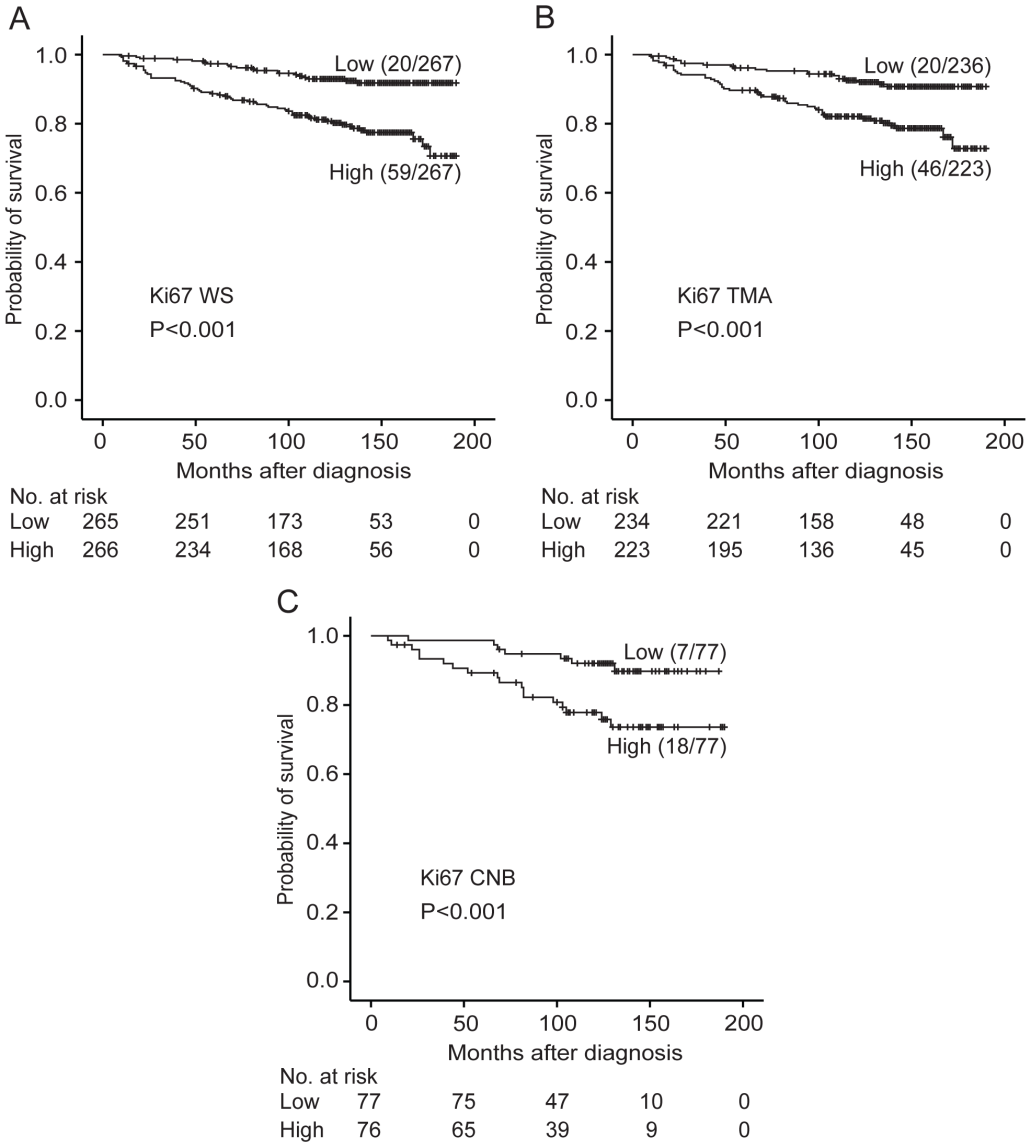
Breast cancer specific survival according to Ki67 expression. Survival curves (Kaplan-Meier) are shown for Ki67 expression on WS (A); TMA (B) and CNB (C). Cut-off points at the median were applied for all specimen categories. The number of events and total number of patients in each group are shown beside the description of each curve. Numbers at risk are presented below each curve.

Univariate analyses displayed significant associations between Ki67-WS and patient survival using a cut-off at the median (Figure 3, see also table S3 in File S1). Further, significant influence of Ki67-WS counts was shown for all cut-points examined (10^th^–90^th^ percentiles, Figure S4). Multivariate survival analyses, after adjustment for basic prognostic indicators including age, tumor size, histologic grade and lymph node status, showed that Ki67, tumor size and nodal stage were independent prognostic factors for breast cancer specific survival (Table 5).

**Table 5:** Multivariate survival analysis (Cox′ proportional hazards method) using different specimen categories.

Variables	*N*	HR	95% CI	*P-*value^a^
**A. Whole sections** (final model; n = 529)				
**Tumor diameter**				
≤2 cm	404			
>2 cm	125	2.3	1.4–3.7	0.001
**Nodal status**				
Negative	387			
Positive	142	3.3	2.0–5.3	<0.001
**Ki67 count** b				
Low, ≤18.3	265			
High,>18.3	264	2.4	1.4–4.1	0.001
**B. Core needle biopsies** (final model; n = 153)				
**Nodal status**				
Negative	112			
Positive	41	4.2	1.9–9.5	0.001
**Ki67 count** b				
Low, ≤12.8	77			
High,>12.8	76	2.8	1.1–6.7	0.024
**C. TMAs** (final model; n = 455)				
**Tumor diameter**				
≤2 cm	346			
>2 cm	109	2.0	1.2–3.5	0.009
**Nodal status**				
Negative	335			
Positive	120	3.5	2.0–6.0	<0.001
**Ki67 count** b				
Low, ≤7.0	236			
High,>7.0	219	2.2	1.3–3.7	0.005

HR, Hazard ratio; CI, confidence interval.

Final models after initial inclusion of age, tumor diameter, histologic grade, nodal status and Ki67.

5 cases (WS), 1 case (CNB) and 4 cases (TMA) were excluded due to missing information on lymph node status.

a Likelihood ratio.

b Cut-off point at the median.

Proliferation by Ki67-CS showed similar but weaker effects on BCSS in univariate analysis (Table S4 in File S1). We further performed survival analyses after excluding the 33 cases with locally advanced disease; the results were similar (data not shown).

Univariate survival analysis of Ki67 in CNB sections showed all examined cut-points above the 40^th^ percentile to be prognostic. Multivariate analyses were performed, adjusting for age, tumor size, histologic grade and nodal status. In the final model, Ki67-CNB and nodal status retained prognostic significance.

For Ki67 in TMA sections, univariate survival analyses demonstrated all examined cut-points above the 10^th^ percentile to be prognostic. In multivariate analysis, including the variables age, tumor size, histologic grade and nodal status, Ki67-TMA showed independent prognostic impact in addition to tumor size and nodal status.

Finally, Ki67 on WS, CNB and TMA (paired cases, n = 137) were included in a multivariate analysis. In this model, only Ki67-WS demonstrated independent prognostic significance. (HR 1.06; (1.02–1.10), *P* = 0.006, Ki67 included as a continuous variable).

### Survival by Ki67 in different molecular subgroups

**Figure 4:**
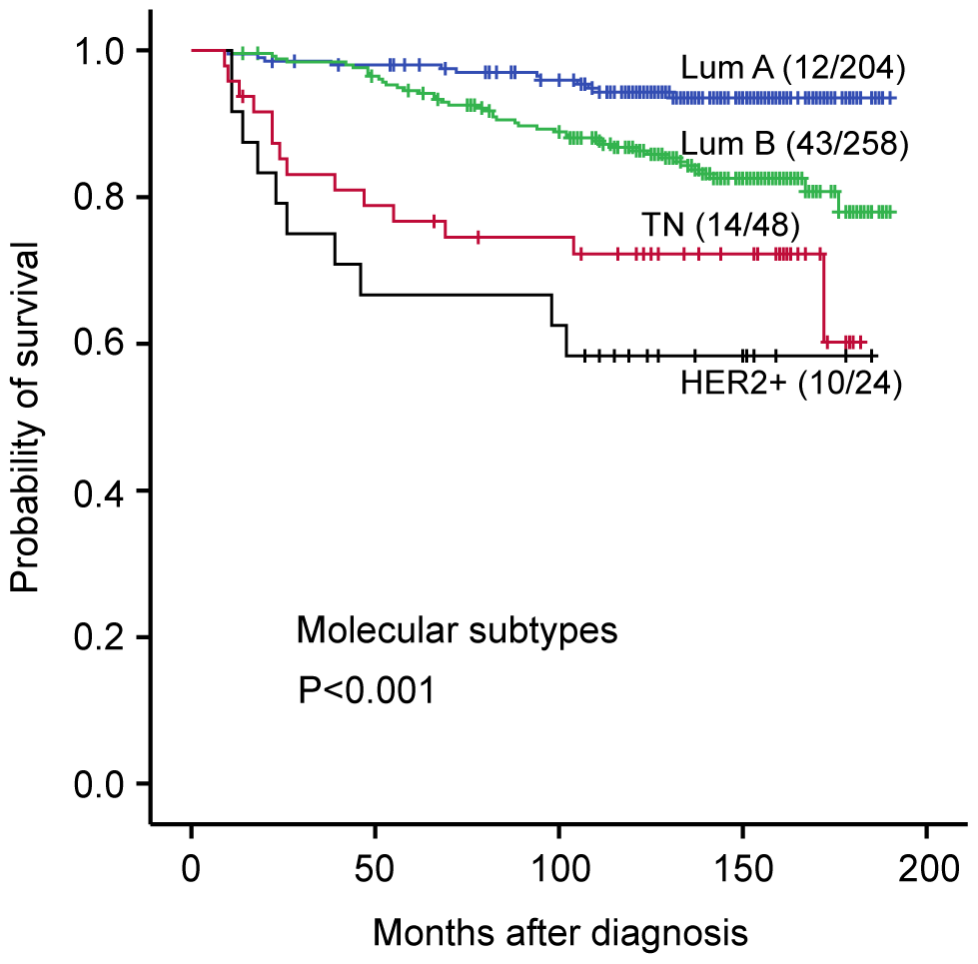
Breast cancer prognosis by molecular subtype. Survival curves (Kaplan-Meier) for breast carcinomas showing an association between molecular subtype and breast cancer specific survival. The Luminal B subgroup includes Luminal/HER2+ cases. For each category, the number of events is given followed by the number of patients.

We also performed subgroup analyses on the complete series stratified by ER and HER2 status and based on Ki67-WS. In the luminal category (including Luminal-HER2+; n = 462), univariate survival analysis revealed a significant association between Ki67 and BCSS (HR 1.03, 95% CI = 1.02 to 1.04; *P*<0.001), also when using two categories with a defined cut-point of 14%, (HR 2.9, 95% CI = 1.5 to 5.5; *P* = 0.001; Figure 4). In multivariate analysis including Ki67 and the basic prognostic variables tumor size, histologic grade and lymph node status, Ki67 retained prognostic significance (together with nodal status and tumor size). Furthermore, by excluding the HER2+ cases and focusing on HR+ breast cancers (n = 412), similar results were obtained (data not shown). In contrast, univariate survival analysis revealed no significant association between Ki67 and outcome within the HER2+/HR- subgroup. By including the HER2+/HR+ cases, the analysis showed prognostic impact of Ki67 (HR 1.027, 95% CI 1.002–1.053; *P* = 0.033). Finally, univariate analysis demonstrated no association between Ki67 and survival in triple negative breast cancer.

## Discussion

It is well documented that tumor cell proliferation by Ki67 expression is strongly associated with breast cancer prognosis [15]. After the suggestion of Ki67 as a predictive marker for adjuvant chemotherapy, observer variation and methodological issues have been increasingly discussed [8], [16]. Some recommendations for Ki67 assessment were presented in 2011, and the lack of systematic comparisons of Ki67 expression levels between tissue microarrays (TMA) and whole sections (WS) was noted [8]. As an example, the Ki67 cut-off point of 14% recommended for treatment decisions by the St Gallen 2011 guidelines was based on data from a series of tissue microarrays combined with gene expression analysis [3], [7]. However, the clinical translation of these findings has not been well documented.

Here, we found a significant difference in proliferation level related to specimen type, with median Ki67 staining values of 18%, 13% and 7% for WS, CNB and TMA samples. These differences might in part be explained by intra-tumor heterogeneity, which is seen both at the morphological and molecular levels [17]–[25].

Studies based on CNB and TMA specimens are challenging as the amount of tissue examined is reduced compared with WS samples. Heterogeneity might especially affect studies using the hot-spot approach, since these areas are often small and might be missed on CNB and TMA sections. Still, prior studies of proliferation markers in breast tumors have shown good statistical correlation between TMA and full sections for Ki67 [26]–[28], and expected associations between Ki67 and clinico-pathologic and molecular features have been reproduced [29]. Also, the use of pre-surgical CNB has been validated for various biomarkers with significant correlation between methods [30]. Good to excellent agreement has been demonstrated for hormone receptors and HER2 status, whereas histologic grade has shown only modest concordance, mainly due to underestimation of mitotic count on CNB specimens [31]–[35]. Some studies on Ki67 have shown good concordance between CNB and WS tissues [36]–[39], whereas others have found only fair to moderate agreement [25], [40], [41]. Notably, even in studies demonstrating a good statistical correlation, there could be marked differences in scores on an individual basis [36]. In our study, a significant proportion of the cases are classified differently given a predetermined threshold and with potential consequences for patient treatment. Importantly, we found that 21% of Luminal A cases on CNB were upgraded to Luminal B on WS specimens, similar to other findings [42].

The subdivision of ER-positive tumors into Luminal A and Luminal B is based on the expression levels of proliferation-related genes among HER2 negative cases. Studies have revealed that proliferation levels are continuous, and sub-classification based on certain cut-points is therefore likely to be arbitrary [43], [44]. Although the 14% cut-off point to separate Luminal A from Luminal B tumors was based on Ki67 expression in TMA samples and established against gene expression profiles, this cut-point showed only a modest sensitivity of 77% and a specificity of 78% in that study [3]. In spite of this, the 14% threshold has been used in research settings as well as in the St Gallen 2011 statement for clinical implementation. Interestingly, the size of the Luminal B subgroup has varied from 8% [45] to 66% [46] in published series. In our study, the 14% cut-off point results in an overestimation of the Luminal B subgroup based on WS specimens, whereas the TMA approach appears to underestimate same group. In the study by Cheang and colleagues, the Luminal B category represents 36% of the HR+/HER2 negative cases [3]. We applied this frequency to our series, and found that the following cut-off points for Ki67 would result in a similar size of the Luminal B-HER2 negative subgroup: 20% (WS), 15% (CNB) and 8% (TMA). Thus, the importance of tissue-specific cut-off points must be considered, for instance when using core needle biopsies and when translating data from TMA-based research to a potential clinical use.

For prognostic purposes, there is no consensus regarding counting area or how many tumor cells should be scored [8]. Although a previous study showed that both the peripheral, central and average Ki67 rates were associated with overall survival [17], two recent studies have revealed that Ki67 has the strongest prognostic impact when counted in hot-spot areas [47], [48]. Notably, using whole sections and hot-spot readings corresponds to what is done for mitotic activity as part of histologic grading. Since prognostic studies have indicated that disease progression is best predicted by Ki67 counted in hot-spot areas, a similar approach should probably be considered for predictive purposes. This must be assessed in carefully designed studies.

Regarding methodology, our study has some limitations, since pre-analytical and analytical variables can not be completely standardized in such retrospective studies [8]. Delayed formalin fixation may result in decreased expression of certain biomarkers [49], although a study of Ki67 found no decrease in expression after 180 minutes delay [50]. Of note, it has been shown that prolonged formalin fixation may cause more extensive masking of antigens, and that not all of this loss can be recovered by antigen retrieval [51], [52]. Further, the TMA technique carries some drawbacks, such as sampling errors and loss of information due to missing tissue cores. Notably, false negative results have been reported for biomarkers studied on TMA sections [53], but it is not known whether this is applicable to Ki67. Regarding ER and PR expression, we used a threshold of 10% for molecular sub-classification according to national guidelines at the time, as compared to the 1% threshold recommended by the present St Gallen guidelines.

In conclusion, tumor cell proliferation as estimated by Ki67 is significantly dependent on specimen category, and our results indicate that specimen-specific cut-off values should be established and validated for clinical use. Furthermore, Ki67 is prognostic over a wide range of cut-off points. For practical purposes, whole sections should be preferred when available, in parallel to the assessment of mitotic count as an integral part of histologic grading. When using hot-spot readings on whole sections, a cut-off point of 20% as a minimum for Ki67 seems to be appropriate at least to predict disease progression. This is also in line with the recent St Gallen 2013 statement [10]. The value of Ki67 as a predictive marker needs to be further studied and validated.

## Supporting Information

File S1 **Supplementary tables.** Table S1. Clinico-pathological features and associations with molecular subtypes of breast cancer. Table S2. Ki67 assessed in hot-spots and cold-spots on WS specimens and associations with histopathological variables. Table S3. Univariate survival analysis according to histopathological variables (Kaplan-Meier method). Table S4. Unadjusted Cox proportional hazards analysis used to estimate the prognostic value of Ki67 expression according to specimen category.
(PDF)

Figure S1 **A.** Ki67 expression scores across specimen category. The median and inter-quartile range of Ki67 is shown according to specimen type. B. Bland-Altman plot is shown for Ki67 expression on whole sections and core needle biopsies. Ki67 difference (WS-CNB) versus average of WS and CNB with 95% limits of agreement (LOA). The mean difference was 2.8% (95% LOA between -22 and 27; *P* = 0.005). **C.** Bland-Altman plot is shown for Ki67 expression on whole sections and TMA. Ki67 difference (WS-TMA) versus average of WS and TMA with 95% LOA. The mean difference was 10% (95% LOA between -10 and 36; *P*<0.001).
(TIF)

Figure S2 **Scatter plots with line of equality illustrating the relationships between counts based on WS, CNB, and TMA specimens.**
(TIF)

Figure S3 **Frequency of cases showing high proliferation when applying a Ki67 cut-off point of 14% to different specimen categories, WS (** ***n*** **= 534), CNB (** ***n*** **= 154), TMA (** ***n*** **= 459).**
(TIF)

Figure S4 **Unadjusted Cox proportional hazards analysis used to estimate the prognostic value of possible Ki67 cut-off points.** The hazard ratio (solid lines) including 95% CI (dashed lines) is shown in dependence of Ki67 cut-off points based on percentiles, with separate plots for WS (A), TMA (B), and CNB (C) specimens.
(TIF)
