# Supplementary material for: Evaluation of Ki67 Expression across Distinct Categories of Breast Cancer Specimens: A Population-Based Study of Matched Surgical Specimens, Core Needle Biopsies and Tissue Microarrays
Source: PLoS One. 2014 Nov 6;9(11):e112121. doi: 10.1371/journal.pone.0112121 (PMC4223011; doi:10.1371/journal.pone.0112121)
Supplement: File S1 — Supplementary tables. Table S1. Clinico-pathological features and associations with molecular subtypes of breast cancer. Table S2. Ki67 assessed in hot-spots and cold-spots on WS specimens and associations with histopathological variables. Table S3. Univariate survival analysis according to histopathological variables (Kaplan-Meier method). Table S4. Unadjusted Cox proportional hazards analysis used to estimate the prognostic value of Ki67 expression according to specimen category. (PDF) [file pone.0112121.s001.pdf]

Table S1 Clinico-pathological features and associations with molecular subtypes of breast cancer

Characteristics	Luminal A[a]		Luminal B[b]		HER2 positive		Triple negative	
	No	(%)	No	(%)	No	(%)	No	(%)
**Tumor diameter**								
≤2 cm	172	84.3	192	74.4	12	50.0	29	60.4
>2 cm	32	15.7	66	25.6	12	50.0	19	39.6
**Histologic grade**								
1	129	63.2	85	32.9	3	12.5	1	2.1
2	70	34.3	134	51.9	7	29.2	15	31.3
3	5	2.5	39	15.1	14	58.3	32	66.7
**Nodal status**[c]								
Negative	163	80.3	178	69.5	10	45.5	36	75.0
Positive	40	19.7	78	30.5	12	54.5	12	25.0
**Histologic type**								
Ductal	159	77.9	227	88.0	20	83.3	41	85.4
Lobular	29	14.2	24	9.3	2	8.3		
Tubular	7	3.4	1	0.4				
Mucinous	9	4.4	6	2.3	1	4.2		
Medullary					1	4.2	3	6.3
Unclassified							4	8.3

[a]Cut-off point 14% used to separate Luminal A from Luminal B tumors
[b]Luminal B includes luminal/HER2+
[c]5 cases with missing information on lymph node status.

Table S2 Ki67 assessed in hot-spots and cold-spots on WS specimens and associations with histopathological variables

	Ki67 WS hot -spot		Ki67 WS cold -spot	
**Variables**	**Median (%)**	***P*-value[a]**	**Median (%)**	***P*-value[a]**
**Tumor diameter**		<0.001		0.146
≤ 2 cm	16.8		12.6	
> 2 cm	28.0		16.0	
**Histologic grade**		<0.001		<0.001
1	12.0		9.5	
2	19.5		13.3	
3	43.7		26.1	
**Nodal status[b]**		0.002		0.025
Negative	16.8		12.4	
Positive	23.3		16.2	
**ER**		<0.001		<0.001
Positive	16.6		11.6	
Negative	42.8		25.6	
**PR**		<0.001		0.005
Positive	16.8		11.6	
Negative	26.2		17.8	
**HER2**		<0.001		0.088
Negative	16.8		12.0	
Positive	32.4		24.2	

[a]Mann-Whitney or Kruskal-Wallis test
[b]5 cases with unknown lymph node status were excluded.

Table S3 Univariate survival analysis according to histopathological variables (Kaplan-Meier method)

Variables	*N*	Events	Estimated survival rates (%) 5 years	10 years	*P*-value[a]
**Tumor diameter**					
≤ 2 cm	405	37	96.0	91.8	
> 2 cm	129	42	83.6	72.1	< 0.001
**Histologic grade**					
1	218	19	96.3	92.5	
2	226	37	94.6	86.4	
3	90	23	81.1	75.4	< 0.001
**Nodal status**[b]					
Negative	387	31	96.3	93.0	
Positive	142	45	84.5	71.7	< 0.001
**ER**					
Positive	451	51	96.6	90.7	
Negative	83	28	73.2	66.9	< 0.001
**PR**					
Positive	377	41	96.8	91.2	
Negative	157	38	83.9	76.9	< 0.001
**HER2**					
Negative	463	62	94.6	88.6	
Positive	71	17	83.1	77.2	0.008
**Ki67**[c]					
Low ≤ 18.3	267	20	97.3	93.0	
High > 18.3	267	59	88.7	81.2	<0.001

[a] Log rank test
[b] 5 cases with unknown lymph node status were excluded.
[c] Ki67 assessed on WS; cut-off point at the median.

Table S4 Unadjusted Cox proportional hazards analysis used to estimate the prognostic value of Ki67 expression according to specimen category.

Variable	Hazard ratio	95% CI	*P*-value*
Ki67-HS whole section	3.2	1.9-5.3	<0.001
Ki67-CS whole section	1.8	1.2-2.9	0.009
Ki67 estimated average	2.8	1.7-4.5	<0.001
Ki67 CNB	3.0	1.3-7.3	0.013
Ki67 TMA	2.7	1.6-4.5	<0.001

Ki67-HS: Ki67 expression assessed in hot-spots; Ki67-CS: Ki67 expression assessed in cold-spots; Est. average: estimated average of Ki67-HS and Ki67-CS, CNB: core needle biopsy; TMA: tissue microarray.
*Likelihood ratio
Ki67 categorized at the median.
