# Supplementary figures and images for: Evaluation of Ki67 Expression across Distinct Categories of Breast Cancer Specimens: A Population-Based Study of Matched Surgical Specimens, Core Needle Biopsies and Tissue Microarrays
Source: PLoS One. 2014 Nov 6;9(11):e112121. doi: 10.1371/journal.pone.0112121 (PMC4223011; doi:10.1371/journal.pone.0112121)

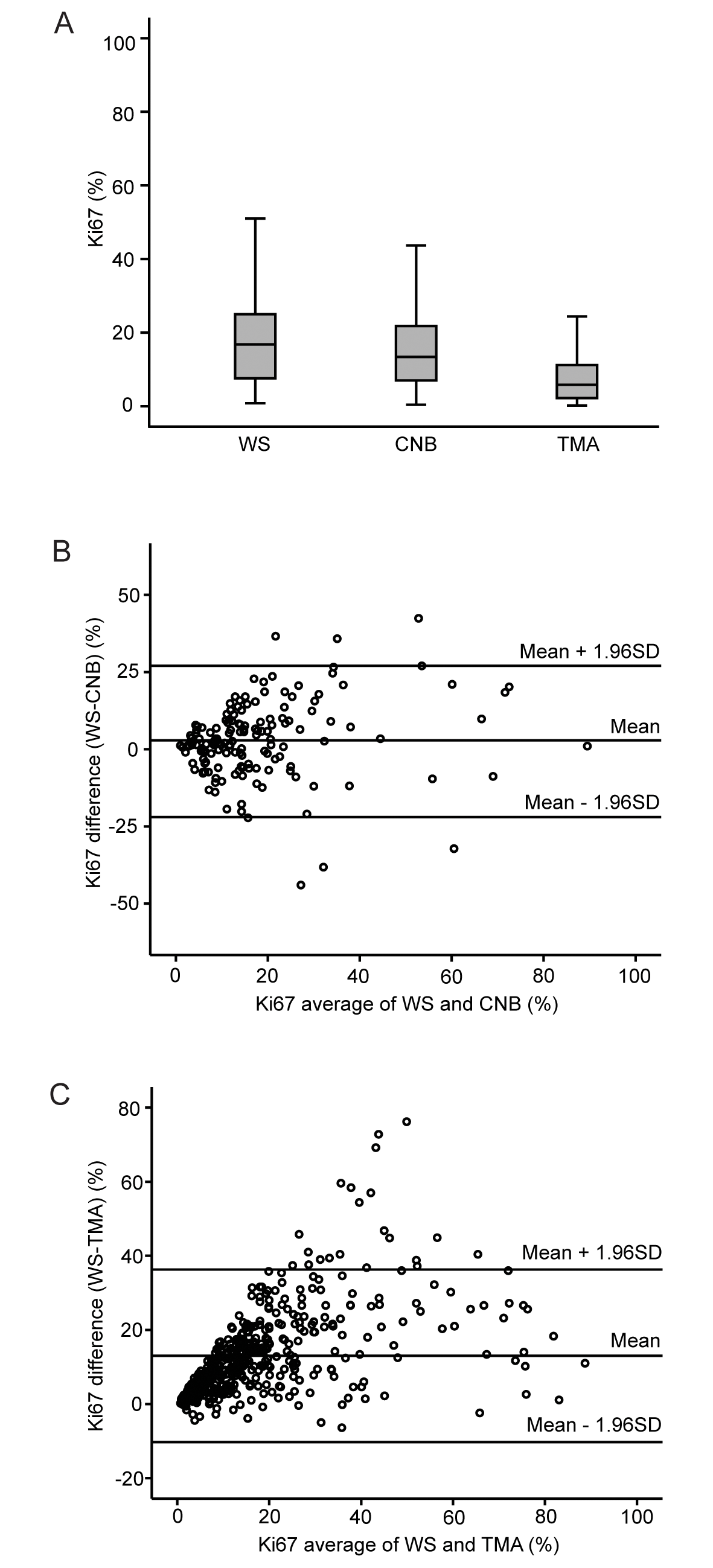

Supplement: Figure S1 — A. Ki67 expression scores across specimen category. The median and inter-quartile range of Ki67 is shown according to specimen type. B. Bland-Altman plot is shown for Ki67 expression on whole sections and core needle biopsies. Ki67 difference (WS-CNB) versus average of WS and CNB with 95% limits of agreement (LOA). The mean difference was 2.8% (95% LOA between -22 and 27; P = 0.005). C. Bland-Altman plot is shown for Ki67 expression on whole sections and TMA. Ki67 difference (WS-TMA) versus average of WS and TMA with 95% LOA. The mean difference was 10% (95% LOA between -10 and 36; P<0.001). (TIF) [file pone.0112121.s002.tif]

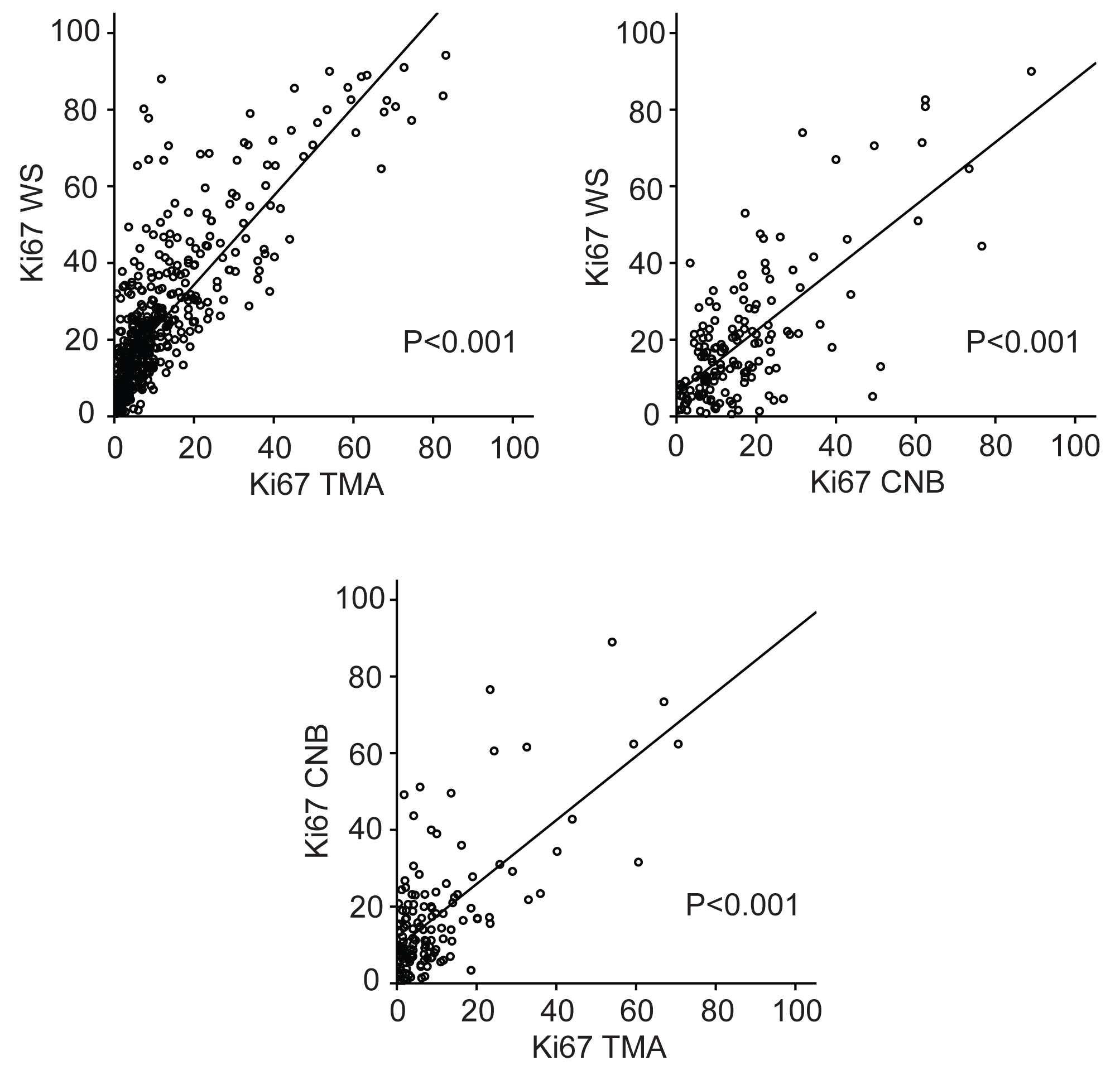

Supplement: Figure S2 — Scatter plots with line of equality illustrating the relationships between counts based on WS, CNB, and TMA specimens. (TIF) [file pone.0112121.s003.tif]

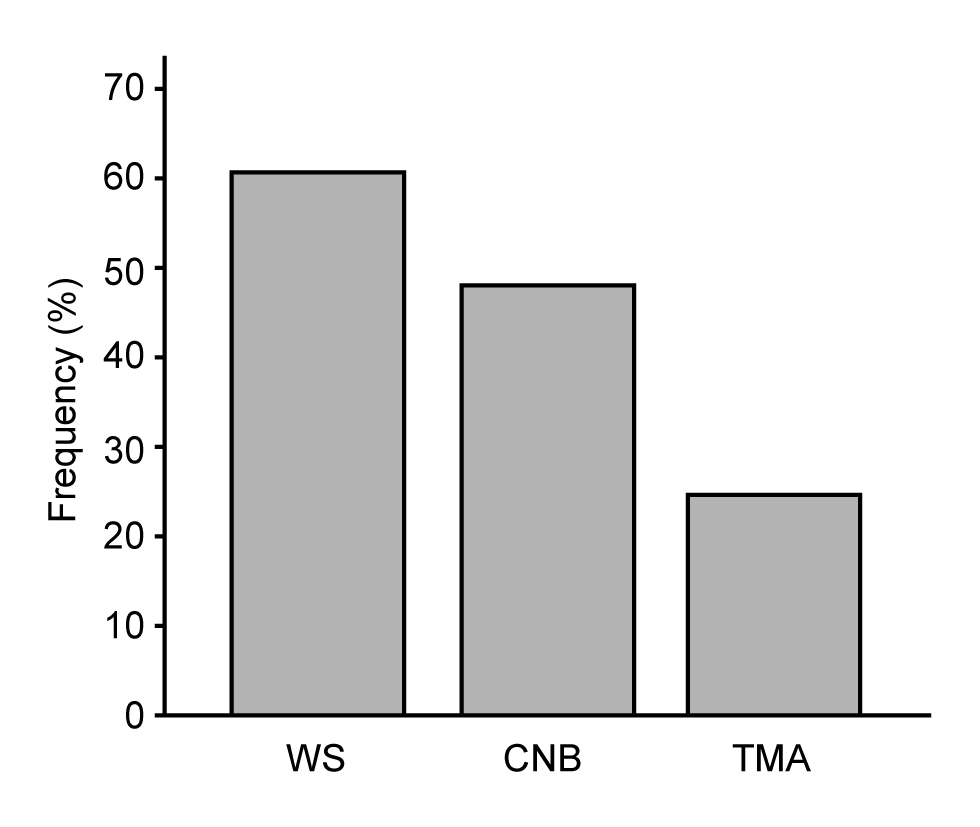

Supplement: Figure S3 — Frequency of cases showing high proliferation when applying a Ki67 cut-off point of 14% to different specimen categories, WS ( n = 534), CNB ( n = 154), TMA ( n = 459). (TIF) [file pone.0112121.s004.tif]

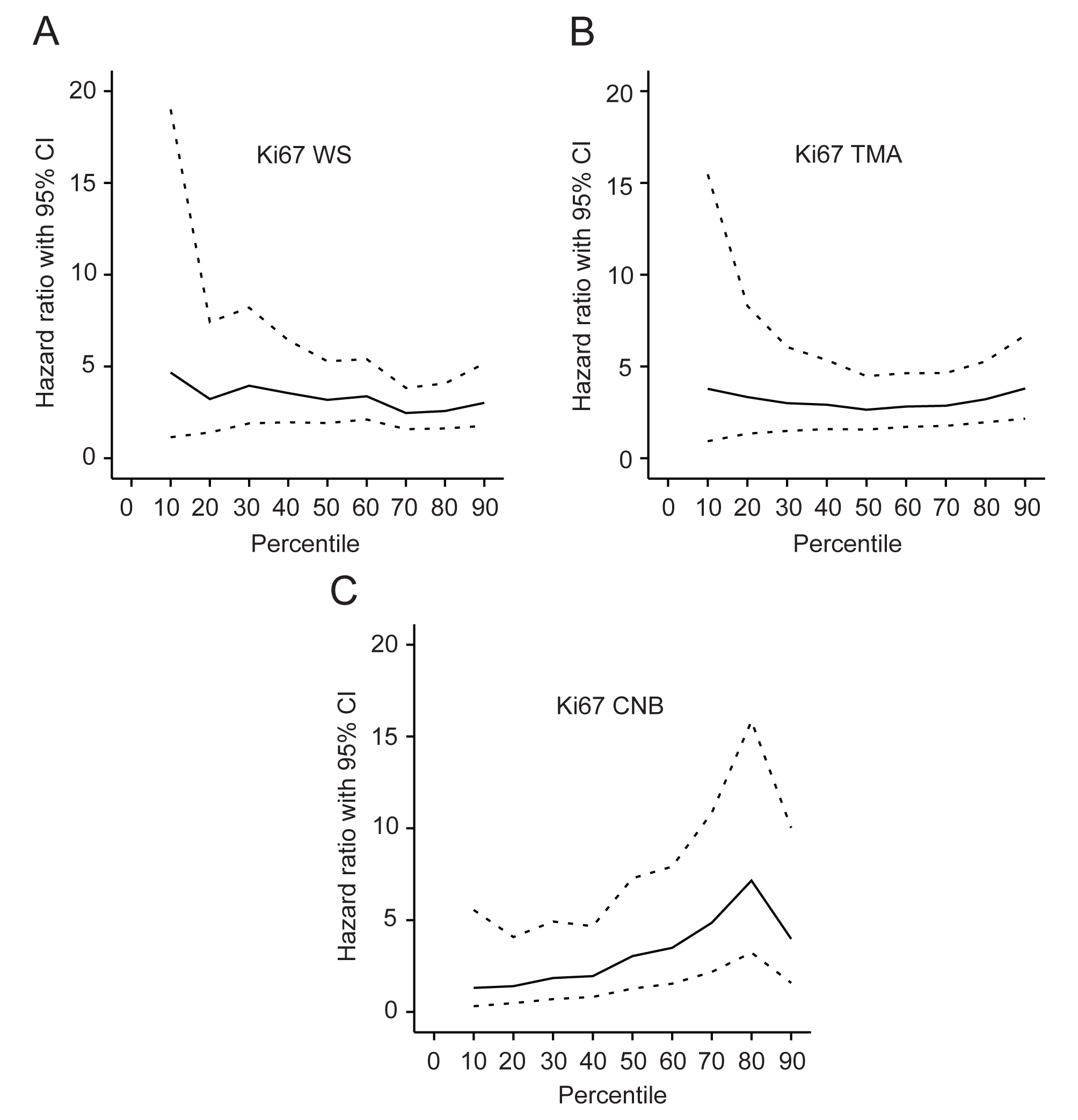

Supplement: Figure S4 — Unadjusted Cox proportional hazards analysis used to estimate the prognostic value of possible Ki67 cut-off points. The hazard ratio (solid lines) including 95% CI (dashed lines) is shown in dependence of Ki67 cut-off points based on percentiles, with separate plots for WS (A), TMA (B), and CNB (C) specimens. (TIF) [file pone.0112121.s005.tif]
